# N-Methyl-D-Aspartate Receptors Antagonist Prevents Secondary Ischemic Brain Injury Associated With Lipopolysaccharide-Induced Sepsis-Like State Presumably *via* Immunomodulatory Actions

**DOI:** 10.3389/fncel.2022.881088

**Published:** 2022-05-20

**Authors:** Golnar Taheri, Maryam Sardari, Dirk M. Hermann, Houri Sepehri

**Affiliations:** ^1^Department of Animal Biology, School of Biology, College of Science, University of Tehran, Tehran, Iran; ^2^Department of Neurology, University Hospital Essen, Essen, Germany

**Keywords:** focal cerebral ischemia, ischemic stroke, neuroinflammantion, neurological deficits, sepsis

## Abstract

Infection is a major reason for poor stroke outcomes, and sepsis is a major cause of stroke-elated deaths. We herein examined whether NMDA receptor blockade, which was reported to exert anti-inflammatory actions, protects against the deleterious consequences of lipopolysaccharide (LPS)-induced sepsis-like state in adult male NMRI mice exposed to transient intraluminal middle cerebral artery occlusion (MCAO). At 24 h post-ischemia, vehicle or *Escherichia coli* LPS (2 or 4 mg/kg) was intraperitoneally administered, whereas 30 min later vehicle or ketamine (10 mg/kg), which is a non-competitive NMDA receptor antagonist, was intraperitoneally applied. Delivery of LPS at a dosage of 4 mg/kg induced a sepsis-like state characterized by a rectal temperature reduction by ∼4.0°C, increased neurological deficits in Clark score, cylinder and open-field tests, increased brain infarct volume and reduced neuronal survival in the previously ischemic tissue. Notably, additional treatment with ketamine (10 mg/kg) significantly attenuated the sepsis-associated rectal temperature reduction by ∼1.5°C, reduced neurological deficits, reduced infarct volume, and promoted neuronal survival. Ketamine alone did not influence infarct volume or neurological deficits. Real-time PCR data analysis showed that *GFAP*, *CD86*, *CD206*, *IL-1*β, and *IL-10* mRNA levels were significantly increased in ischemic brains of LPS-treated compared with vehicle-treated mice. Additional treatment with ketamine significantly decreased *IL-1*β and *IL-10*, but not *GFAP*, *CD86*, and *CD206* mRNA levels. Our data show that ketamine at a dose that on its own does not confer neuroprotection reverses the adverse effects of LPS-induced sepsis-like state post-ischemia, presumably *via* immunomodulatory actions.

## Introduction

Despite recent advances in acute ischemic stroke management, stroke is still ranked as the second leading cause of death and one of the main causes of long-term disability worldwide (Johnson et al., 2016). Stroke-associated systemic inflammatory responses are typically followed by immune suppression, aiming to protect the brain from further inflammatory damage (Faura et al., 2021). As a bystander effect, immune suppression increases the susceptibility of patients to infections. Approximately 30% of patients develop infections shortly after stroke (Aslanyan et al., 2004), among which pneumonia and urinary tract infections are most common. Post-ischemic infections augment the severity of stroke symptoms and mortality rates in stroke patients (Westendorp et al., 2011). Given this fact, there is a noteworthy need to identify and mitigate factors compromising neurological recovery once a stroke has occurred.

Lipopolysaccharide (LPS) is a major component of the outer membrane of gram-negative bacteria, such as *Escherichia coli*. LPS activates pro-inflammatory signals through toll-like receptor-4 (TLR-4), which in the brain is mainly expressed by innate brain immune cells, namely in microglial cells (Hines et al., 2013). It is well documented that systemic administration of the LPS results in blood-brain barrier disruption (Cheng et al., 2018), besides induction of pro-inflammatory phenotype of the microglia cells, leading to an increase in pro-inflammatory cytokines production causing neuronal injury (An et al., 2020). These inflammatory responses are accomplished by long-lasting disturbances of endogenous neurogenesis, neuroplasticity, and cognitive performance (Anderson et al., 2015). Due to these features, LPS delivery is considered an attractive model of gram-negative sepsis. Post-ischemic delivery of LPS increases peripheral immune cells infiltration to the brain, induces microglial over-activation, and exacerbates ischemic brain injury in the mouse intraluminal middle cerebral artery occlusion (MCAO) model (Sardari et al., 2020b). Although the LPS exposure acts as an inflammatory stimulus, some studies with early post-ischemic LPS delivery have also reported neuroprotective effects of LPS in mice exposed to intraluminal MCAO (Sardari et al., 2020a). The animal species, timing, and dosing of LPS decisively influence ischemic brain tissue responses.

Following an ischemic event, the cellular release and extracellular concentration of glutamate, the most abundant excitatory neurotransmitter of CNS, increases, resulting in secondary brain injury (Kirdajova et al., 2020). Moreover, it has been reported that this type of excitotoxic injury is at least partly mediated mainly *via* ionotropic N-methyl-D-aspartate receptors (NMDA) (Wu and Tymianski, 2018). Given the pivotal role of the NMDA receptor in excitotoxicity, one of the therapeutic approaches is to block NMDA receptors. Ketamine, a non-competitive NMDA receptor antagonist, is a potent antidepressant, analgesic, and sedative drug widely used in clinical studies (Gao et al., 2016). Subanesthetic doses of ketamine were shown to decrease the severity of ischemic injury by interfering with brain inflammatory responses. In rats, ketamine suppressed LPS-induced TNF-α, IL-6, and IL-8 levels (Ji et al., 2019). In mice exposed to MCAO, ketamine reduced ischemic injury and behavioral neurological deficits (Xiong et al., 2020). Hence we asked whether the NMDA receptor ketamine might mitigate injury-promoting consequences of LPS-induced sepsis-like state in the mouse intraluminal MCAO model.

## Materials and Methods

### Animal Care and Legal Issues

A total of 62 adult male NMRI mice weighing 25–30 g were used in this study. Mice were housed in the University of Tehran animal facility in cohorts of 4 animals per cage and kept under standard animal room conditions (ambient temperature 20 ± 2°C, 12/12-h light-dark cycle). The mice had free access to food and water. Before surgery, the animals were allowed to adapt to the laboratory conditions and handled for 5 min per day during the 1-week acclimatization period by the experimenter who later performed the behavioral tests. The experiments were performed during the light phase of the cycle between 08:00 h and 14:00 h. All procedures for the treatment of animals were approved by the Research and Ethics Committee of the School of Biology at the University of Tehran (IR.UT.SCIENCE.REC.1400.001).

### Ischemic Stroke Model

Male NMRI mice (8–9 weeks; 25–30 g) anesthetized with ketamine (50 mg/kg) and xylazine (5 mg/kg) were exposed to 15 min left-sided intraluminal middle cerebral artery occlusion (MCAO). Rectal temperature was maintained between 36.5 and 37.0°C using a feedback-controlled heating system (Borj Sanat Company, Tehran, Iran). The left common and external carotid arteries were isolated and ligated, and the internal carotid artery was temporarily clipped. A nylon filament coated with a silicon tip with 0.21 mm diameter (Cat No., 6021PK5Re, Doccol Co., Redlands, United States) was introduced through a small incision into the common carotid artery and advanced to the carotid bifurcation for MCAO. Reperfusion was initiated by monofilament removal. In non-ischemic sham-operated mice, a midline neck incision was made during anesthesia, *via* which the CCA was exposed. Immediately after surgery, wounds were carefully sutured, and animals were treated using topical administration of lidocaine gel 2% on the sutured area for pain relief.

### Drug Treatment and Animal Groups

*Escherichia coli* lipopolysaccharide (LPS; 2 or 4 mg/kg in normal saline; 0111: B4; Sigma, Deisenhofen, Germany) and ketamine (10 mg/kg in normal saline; Bremer Pharma, Germany) were intraperitoneally administered 30 min apart at 24 h post-ischemia (hpi). Control animals received the vehicle (normal saline) on each occasion.

The first set of mice exposed to MCAO was treated with vehicle or LPS (2 or 4 mg/kg) at 24 hpi and with vehicle or ketamine (10 mg/kg) 30 min later. These mice received detailed assessments of body weight, rectal temperature and neurological deficits. The mice were deeply anesthetized at 96 hpi, and brains were collected for 2,3,5-triphenyl tetrazolium chloride (TTC) staining (*n* = 6 animals/group).

The second set of mice, which were exposed to MCAO or sham surgery, was treated with vehicle, LPS (4 mg/kg) combined with vehicle, or LPS (4 mg/kg) combined with ketamine (10 mg/kg) at 24 hpi. These mice were sacrificed at 96 hpi for real-time quantitative polymerase chain reaction (qPCR) studies (four dpi; each *n* = 3 animals/group). Brains were taken from these mice, in which *GFAP*, *CD86*, *CD206*, *IL-1*β, and *IL-10* mRNAs were measured by qPCR.

The third set of mice, which were exposed to MCAO or sham surgery, were treated with vehicle, LPS (4 mg/kg) combined with vehicle, or LPS (4 mg/kg) combined with ketamine (10 mg/kg) at 24 hpi. These mice were sacrificed at 96 hpi for Nissl staining (*n* = 5 animals/group).

### Neurological Score and Behavioral Tests

#### Clark Score

The Clark score consists of two subscores capturing general and focal neurological deficits (Clark et al., 2016; Sardari et al., 2020b). General and focal deficits were evaluated at baseline, at 3, 6, and 24 hpi before LPS-induced sepsis, and at 27, 30, 48, 72, and 96 hpi (that is, at 3, 6, 24, and 72 h) after LPS-induced sepsis.

#### Cylinder Test

Mice were put in a transparent cylinder (diameter: 20 cm, height: 40 cm). The number of forepaw contacts to the cylinder wall was counted. Animals were monitored as long as they performed 20 moves, completely separating forelimbs from the floor, contacting one or both to the cylinder wall. In groups showing a decrease in locomotor activity, the timer was set for 6 min (Roome and Vanderluit, 2015; Gil et al., 2018). The score of the cylinder test in this study was calculated as the percentage of impaired forelimb relative to total forelimb uses (Cenci and Lundblad, 2005). The cylinder test was performed at baseline (that is, before MCAO), 24, 48, and 96 hpi.

#### Open-Field Test

Spontaneous locomotor activity was monitored using a computerized open-field apparatus (Borj Sanat Co., Tehran, Iran). The apparatus consisted of a plexiglass open-field apparatus (40 cm) equipped with photobeam beam arrays (four beams on two sides). Mouse movements caused interruptions of infrared photobeams, which were recorded automatically by a counter connected to the apparatus for subsequent analysis. Locomotor activity was recorded over 5 min (Zhao B. et al., 2020). The open-field test was conducted at baseline, 24, 48, and 96 hpi.

#### Elevated Body Swing Test

The elevated body swing test was used for the investigation of asymmetrical motor behavior. Mice were held by the root of their tail and raised 10 cm above the testing surface. The initial swing direction, determined as turning the upper body > 10° to either side, was recorded in 20 trials. The number of turns in each (left or right) direction was recorded for each mouse (Azedi et al., 2019). The elevated body swing test was performed at baseline, 24, 48, 72, and 96 hpi.

### Analysis of Brain Infarcts and Neuronal Survival

At 96 hpi, infarct volume and brain edema were assessed by TTC staining in 2-mm thick coronal brain slices. For analysis of infarct areas, non-lesioned tissue of the ischemic hemisphere was outlined and subtracted from that of the contralateral hemisphere. Subsequently, infarct volume was calculated by integrating infarct areas at rostrocaudal brain levels. Brain edema was evaluated as a relative increase of ipsilateral versus contralateral brain volume.

Furthermore, twenty-μm-thick coronal brain sections were stained with cresyl violet, labeling Nissl substance in surviving neurons. Sections were imaged on a Zeiss Axioplan fluorescence microscope with 40X magnification. Medium-sized surviving neurons were counted in a blinded way in three defined regions of interest (ROI) measuring 1151.50 μm × 1151.50 μm in the ischemic and contralateral non-ischemic striatum. Of the values determined in the ischemic and contralateral striatum, the percentage of surviving neurons was calculated.

### Real-Time qPCR

Messenger RNA (mRNA) was extracted from the whole ischemic brain hemispheres using an RNeasy Mini Kit (Qiagen, Hilden, Germany). mRNA was converted to cDNA using a high-capacity RNA-to-cDNA kit (Thermo Fisher Scientific, Langenselbold, Germany). Real-time qPCR was performed in a StepOnePlus real-time PCR system using primers selected by the PubMed primer-BLAST tool (Table 1).^1^ The efficiency of these primers had been confirmed in melting curves. Glyceraldehyde 3-phosphate dehydrogenase (GAPDH) was used as a housekeeping gene; the brain tissue from sham mice served as control. Results were quantified using the 2^–ΔΔ*Ct*^ method. PCR was performed in triplicate, of which mean values were formed for each mouse.

**TABLE 1:** List of primers.

Accession number	Gene	Forward primer (5′–3′)	Reverse primer (5′–3′)	Product (bp)
NM_001131020.1	GFAP	GGGGCAAAAGCACCAAAGAAG	GGGACAACTTGTATTGTGAGCC	76
NM_019388.3	CD86	GCTCTTACTGACTGGCATGAG	GCTCTTACTGACTGGCATGAG	106
NM_001106123.2	Mrc1 (CD206)	GATTGACCAGTTCCTTGACCT	AACACATTCCAGATTCTCCCA	197
NM_008361.4	IL-1β	GCTCTTACTGACTGGCATGAG	CCGACAGCACGAGGCTTT	76
NM_010548.2	IL-10	GCTCTTACTGACTGGCATGAG	GCTCTTACTGACTGGCATGAG	105
NM_017008.4	GAPDH	GGAGAAACCTGCCAAGTATG	AAGAATGGGAGTTGCTGTTG	131

### Statistical Analysis

Body weight, rectal temperature, neurological score, and behavioral tests were analyzed by two-way repeated-measurement analysis of variance (ANOVA) followed by Tukey’s tests. For dissecting effects of LPS and ketamine from each other, two-way ANOVAs followed by Tukey’s tests were also performed for individual time-points. Infarct volume, edema volume, neuronal survival, and qRTPCR data were analyzed by one-way ANOVA followed by Tukey’s tests. Body weight, behavioral data, and qRTPCR data were presented as mean ± SEM. Infarct volume and edema volume data, which are particularly prone to outliers, were presented as mean ± interquartile range box-blots with minimum/maximum data as whiskers. Significance thresholds were set at *p* < 0.05. Calculations were performed using PRISM version 9.

## Results

### Effects of Lipopolysaccharide and Ketamine on Post-ischemic Body Weight, Rectal Temperature, General and Focal Neurological Scores

**FIGURE 1:**
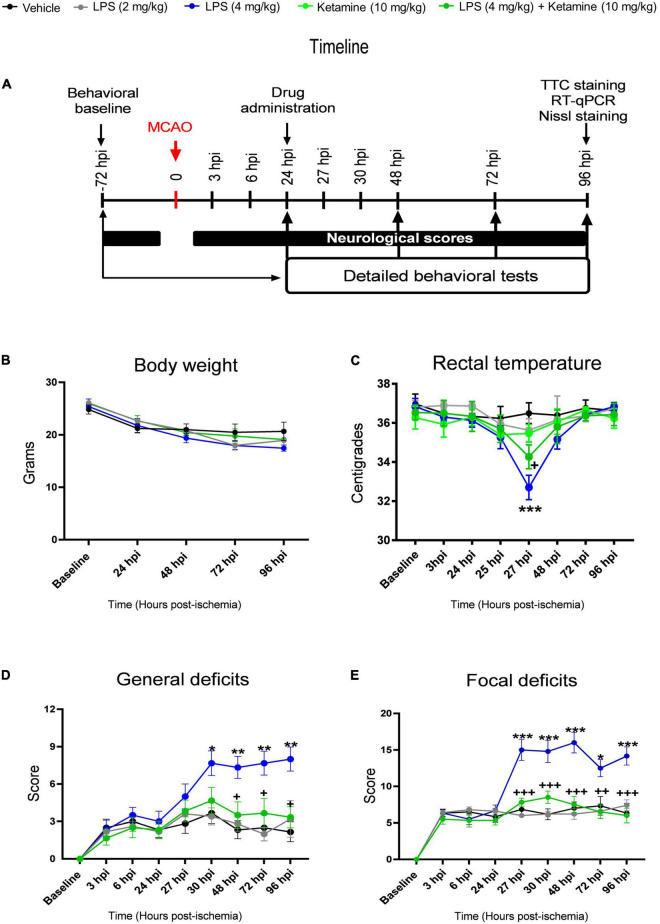
Effects of LPS and ketamine on post-ischemic body weight, rectal temperature, general and focal neurological scores. **(A)** Timeline of the experimental procedure, **(B)** body weight, **(C)** rectal temperature, as well as **(D,E)** general and focal neurological deficits assessed by the Clark score in mice exposed to transient intraluminal middle cerebral artery occlusion (MCAO) which were intraperitoneally injected with vehicle or LPS (2 or 4 mg/kg) at 24 h post-ischemia (hpi). 30 min later, vehicle or ketamine (10 mg/kg) was intraperitoneally administered in mice receiving vehicle or LPS (4 mg/kg). Note that LPS at 4 mg/kg, but not 2 mg/kg, induced hypothermia at 27 hpi (that is, 3 h after LPS injection), indicative of a sepsis-like state, and exacerbated general and focal neurological deficits in the Clark score. Ketamine attenuated the LPS-induced rectal temperature reduction and reversed the general and focal neurological deficits, whereas ketamine alone did not influence rectal temperature or neurological scores. Data are mean ± SEM values (*n* = 6 animals/group). *P < 0.05; **P < 0.01; ***P < 0.001 compared with ischemic vehicle; ^+^*P* < 0.05; ^++^*P* < 0.01; ^+++^*P* < 0.001 compared with ischemic LPS (4 mg/kg).

Vehicle or LPS (2 or 4 mg/kg) were i.p. injected 24 h post-ischemia (hpi). Thirty minutes later, vehicle or ketamine (10 mg/kg) were i.p. applied according to the timeline shown in Figure 1A. This ketamine dose was subanesthetic, it did not have any relevant effect on the behavior of the mice. Post-MCAO, body weight decreased by ∼15% in LPS-treated compared to vehicle-treated mice (Figure 1B). Repeated measurement ANOVA revealed that body weight did not differ significantly between groups [treatment effect: *F*(4, 25) = 1.24, *P* > 0.05; treatment × time interaction: *F*(16, 100) = 1.74, *P* > 0.05].

Rectal temperature measurements revealed that LPS induced hypothermia within 3 h (that is, at 27 hpi) (Figure 1C). At this time-point, rectal temperature decreased by ∼4.0°C, indicative of LPS-induced sepsis-like state. Ketamine significantly attenuated the LPS sepsis-associated temperature reduction by ∼1.5°C (Figure 1C). Repeated measurement ANOVA confirmed that ketamine decreased the temperature reduction in LPS-treated mice [treatment effect: *F*(4, 10) = 4.38, *P* < 0.05; treatment × time interaction: *F*(28, 70) = 4.47, *P* < 0.001]. Ketamine alone did not influence rectal temperature measurements (Figure 1C).

Focal cerebral ischemia induced distinct general and focal neurological deficits, as revealed by the Clark score (Figures 1D,E). LPS at 4 mg/kg significantly increased both general and focal deficits (Figures 1D,E). Interestingly, ketamine prevented the LPS-induced general and focal neurological deficits (Figures 1D,E). Repeated measurement ANOVA confirmed that the LPS-induced general [treatment effect: *F*(4, 24) = 4.69, *P* < 0.001; treatment × time interaction: *F*(32, 192) = 5.27, *P* < 0.001] and focal [treatment effect: *F*(4, 23) = 12.08, *P* < 0.001; treatment × time interaction: *F*(32, 184) = 8.58, *P* < 0.001] neurological deficits were indeed reversed by ketamine. Notably, ketamine alone did not influence general or focal neurological deficits in ischemic mice (Figures 1D,E).

### Effects of Lipopolysaccharide-Induced Sepsis and Ketamine on Post-ischemic Motor-Coordination Deficits

**FIGURE 2:**
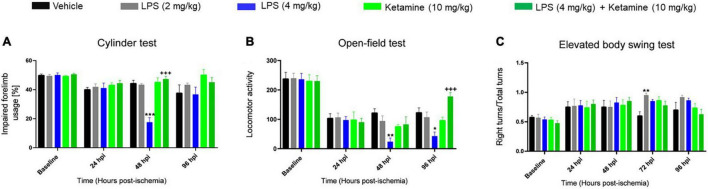
Effects of LPS-induced sepsis and ketamine on post-ischemic motor-coordination deficits. Motor-coordination performance evaluated by **(A)** cylinder, **(B)** open-field, and **(C)** elevated body swing tests of mice exposed to transient MCAO followed by delivery of vehicle or LPS (2 or 4 mg/kg) at 24 hpi. 30 min later, vehicle or ketamine (10 mg/kg) was injected in mice receiving vehicle or LPS (4 mg/kg). Note that LPS-induced sepsis (4 mg/kg) significantly impaired motor-coordination and spontaneous locomotor activity evaluated by the cylinder and open-field tests. Ketamine reversed the LPS-induced motor-coordination deficits, while ketamine alone had no effect. Data are mean ± SEM values (*n* = 6 animals/group). *p < 0.05; **P < 0.01; ***P < 0.001 compared with ischemic vehicle; ^+++^*P* < 0.001 compared with ischemic LPS (4 mg/kg).

We next performed more detailed assessments using a battery of motor-coordination tests. In the cylinder test, a test of forelimb coordination, LPS at 4 mg/kg significantly decreased contralateral forelimb usage of ischemic mice at 48 hpi (Figure 2A). Add-on treatment with ketamine restored contralateral forelimb usage at 48 hpi (Figure 2A). Ketamine alone did not affect contralateral forelimb usage (Figure 2A). As a consequence of the transient nature of this effect, repeated measurement AVOVA revealed no significant treatment [*F*(4, 23) = 1.07, *P* > 0.05] or treatment × time interaction [*F*(12, 69) = 0.67, *P* > 0.05] effects.

LPS at 4 mg/kg significantly decreased spontaneous locomotor activity in the open-field test at 48 and 96 hpi (Figure 2B). Ketamine reversed the LPS-induced locomotor activity reduction but did not influence locomotor activity when administered alone (Figure 2B). This effect was significant in repeated measurement ANOVA [treatment effect: *F*(4, 22) = 1.67, *P* < 0.05; treatment × time interaction: *F*(12, 66) = 2.81, *P* < 0.05].

Additionally, LPS at 2 mg/kg but not 4 mg/kg increased the number of right turns in the elevated body swing test at 72 hpi (Figure 2C). Ketamine did not significantly affect the number of right turns, neither when administered alone nor combined with LPS (Figure 2C). Repeated measurement AVOVA revealed no significant treatment [*F*(4, 16) = 1.67, *P* > 0.05] or treatment × time interaction [*F*(16, 64) = 0.89, *P* > 0.05] effect.

### Effects of Lipopolysaccharide-Induced Sepsis and Ketamine on Infarct Volume and Brain Edema

**FIGURE 3:**
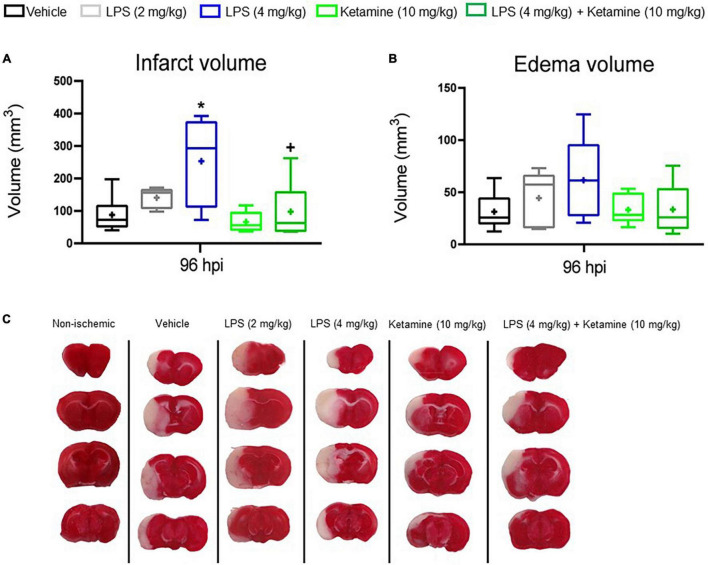
Effects of LPS-induced sepsis and ketamine on infarct volume and brain edema. **(A)** Infarct volume and **(B)** brain edema volume assessed by TTC staining in the brains of mice exposed to transient MCAO followed by vehicle or LPS (2 or 4 mg/kg) delivery at 24 hpi. 30 min later, vehicle or ketamine (10 mg/kg) was injected in mice receiving vehicle or LPS (4 mg/kg). **(C)** Representative TTC-stained coronal brain sections are also shown at 96 hpi (that is, at 72 h after LPS.induced sepsis). Note that LPS-induced sepsis (4 mg/kg) significantly increased infarct volume, which was reversed by ketamine. Ketamine alone had no effect. Data are medians (lines inside boxes)/means (crosses inside boxes) ± interquartile ranges (IQR; boxes) with minimum/maximum values as whiskers (*n* = 6 animals/group). *P < 0.05 compared with the ischemic vehicle; ^+^*P* < 0.05 compared with ischemic LPS (4 mg/kg).

To evaluate the effect of LPS and ketamine on infarct and edema volume, TTC staining was performed. LPS (4 mg/kg) increased infarct volume at 96 hpi, whereas additional treatment with ketamine reversed this action. Ketamine alone did not affect infarct volume (Figures 3A,C). Brain edema did not differ between groups (Figures 3B,C).

### Effects of Lipopolysaccharide and Ketamine on Post-ischemic Neuronal Survival

**FIGURE 4:**
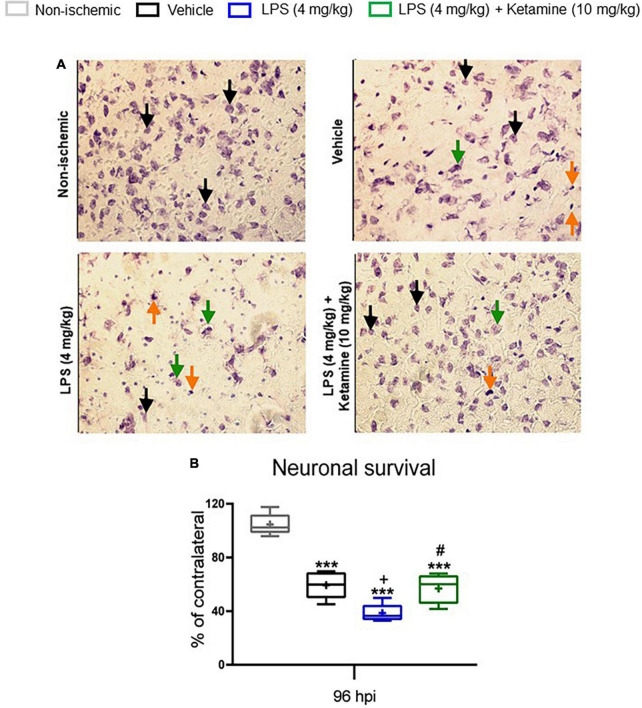
Effects of LPS and ketamine on post-ischemic neuronal survival. **(A)** Neuronal survival assessed by Nissl staining in the ischemic striatum, i.e., the core of the middle cerebral artery territory, of mice exposed to sham surgery or transient MCAO. The ischemic animals were treated with vehicle or LPS (4 mg/kg) at 24 hpi followed by vehicle or ketamine (10 mg/kg) 30 min later. Mice were sacrificed at 96 hpi. The density of surviving neurons in the ischemic striatum was expressed as percentage of the density of neurons in the contralateral non-ischemic striatum. **(B)** Note that transient MCAO markedly reduced neuronal density in the ischemic striatum. LPS-induced sepsis further decreased neuronal density, whereas additional ketamine increased neuronal survival. Data are medians (lines inside boxes)/means (crosses inside boxes)_interquartile ranges (IQR; boxes) with minimum/maximum values as whiskers (n D 5 animals/group). In **(A)** surviving neurons, presumably apoptotic cells and necrotic cells were labeled with black, orange and green arrows, respectively. Only medium-sized surviving neurons with intact shape were counted. ***P < 0.001 compared with non-ischemic sham; ^+^*P* < 0.05 compared with ischemic vehicle; ^#^*P* < 0.05 compared with ischemic LPS. Scale bar: 100 mm.

Next, Nissl staining was performed to analyze surviving neurons in the ischemic striatum, the core of the middle cerebral artery territory. Compared with sham-operated mice not exposed to MCAO, the percentage of surviving neurons was considerably decreased in vehicle-treated ischemic mice (Figure 4). LPS further decreased the percentage of surviving neurons compared with vehicle-treated ischemic mice (Figure 4). Additional treatment with ketamine significantly increased neuronal survival in LPS-treated mice (Figure 4).

### Effects of Lipopolysaccharide and Ketamine on Post-ischemic GFAP, CD86, and CD206 Expression

**FIGURE 5:**
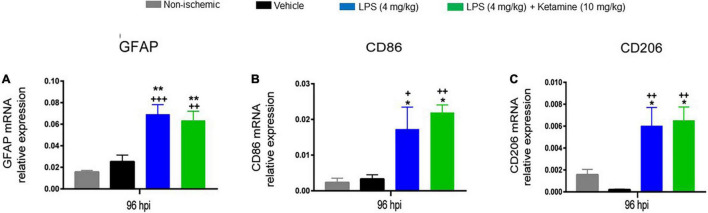
Effects of LPS and ketamine on post-ischemic *GFAP*, *CD86*, and *CD206* expression. Expression of **(A)** *GFAP*, **(B)** *CD86*, and **(C)** *CD206* mRNAs in the ischemic brain tissue of mice exposed to sham surgery or transient MCAO, which were treated with vehicle or LPS (4 mg/kg) at 24 hpi followed by vehicle or ketamine (10 mg/kg) 30 min later. Gene expression was assessed at 96 hpi by RT-PCR. Note that LPS-induced sepsis significantly increased *GFAP*, *CD86*, and *CD206* mRNA levels. Ketamine did not influence *GFAP*, *CD86*, and *CD206* mRNAs. Data are mean ± SEM values (*n* = 3 animals/group). *P < 0.05; **P < 0.01 compared with non-ischemic sham surgery; ^+^*P* < 0.05; ^++^*P* < 0.01; ^+++^*P* < 0.001 compared with ischemic vehicle.

We subsequently asked how LPS and ketamine influenced glial responses in the ischemic brain. For this reason, we conducted qPCR studies for the mRNAs of the astrocytic marker *GFAP*, the pro-inflammatory microglia/macrophage marker CD86, and the anti-inflammatory microglia/macrophage marker CD206. One-way ANOVA revealed that LPS (4 mg/kg) significantly increased *GFAP*, *CD86*, and *CD206* mRNA levels post-MCAO (Figure 5). Additional treatment with ketamine did not influence *GFAP*, *CD86*, and *CD206* mRNA levels (Figure 5).

### Effects of Lipopolysaccharide and Ketamine on Post-ischemic IL-1β and IL-10 Expression

**FIGURE 6:**
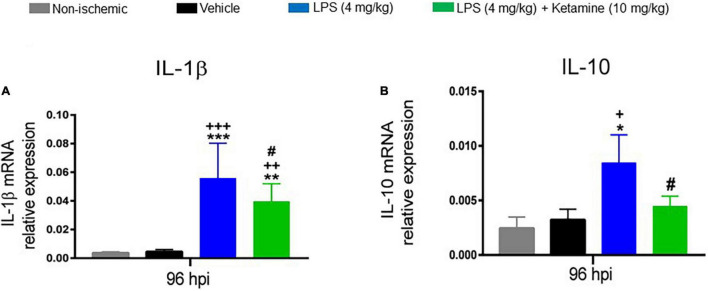
Effects of LPS and ketamine on post-ischemic *IL-1*β and *IL-10* expression. Expression of **(A)** *IL-1*β and **(B)** *IL-10* mRNAs in the ischemic brain tissue of mice exposed to sham surgery or transient MCAO, which were intraperitoneally treated with vehicle or LPS (4 mg/kg) at 24 hpi followed by vehicle or ketamine (10 mg/kg) 30 min later. Gene expression was assessed at 96 hpi by RT-PCR. Note that LPS-induced sepsis significantly increased *IL-1*β and *IL-10* mRNA levels. Ketamine partly reversed the elevated *IL-1*β and *IL-10* mRNA levels. Data are mean ± SEM values (*n* = 3 animals/group). *p < 0.05; **P < 0.01; ***P < 0.001 compared with non-ischemic sham surgery; ^+^*P* < 0.05; ^++^*P* < 0.01; ^+++^*P* < 0.001 compared with ischemic vehicle; ^#^*P* < 0.05 compared with ischemic LPS.

We finally asked how LPS or ketamine influenced the mRNA levels of the pro-inflammatory cytokine *IL-1*β and the anti-inflammatory cytokine *IL-10* as assessed by qPCR. One-way ANOVA revealed that the *IL-1*β and *IL-10* mRNA levels were significantly increased by LPS (4 mg/kg) (Figure 6). Additional treatment with ketamine reduced the elevated *IL-1*β and *IL-10* mRNA levels (Figure 6). These results suggested that ketamine attenuated both the pro-inflammatory and anti-inflammatory cytokines responses induced by LPS.

## Discussion

In agreement with our previous study on C57BL/6 mice using a very similar model of LPS-induced sepsis-like state (Sardari et al., 2020b), we here report in NMRI mice that post-ischemic delivery of LPS (2 or 4 mg/kg) dose-dependently induced a sepsis-like state characterized by rectal hypothermia. Very similar to the previous study (Sardari et al., 2020b), LPS-induced sepsis increased neurological deficits evaluated by Clark score, cylinder, and open-field tests, increased infarct volume evaluated by TTC stainings and decreased neuronal survival evaluated by Nissl stainings. Interestingly, additional treatment with the non-competitive NMDA receptor antagonist ketamine (10 mg/kg) significantly attenuated the sepsis-associated hypothermia, reversed the LPS-induced neurological deficits, reduced infarct volume and increased neuronal survival. Importantly, ketamine (10 mg/kg) alone did not influence neurological deficits or infarct volume. Mechanistically, increased expression of pro-inflammatory and anti-inflammatory markers *CD86*, *CD206*, *IL-1*β, and *IL-10* mRNAs was noted in the ischemic brains of LPS-treated mice. Additional treatment with ketamine reduced the elevated *IL-1*β and *IL-10* mRNA levels.

Previously, intraperitoneal LPS administration at 72 hpi increased infarct volume and brain atrophy and increased neurological deficits evaluated by tight rope tests in C57BL/6 mice exposed to intraluminal MCAO (Sardari et al., 2020b). In Sprague Dawley rats, intraperitoneal LPS injection (268 μg/kg) immediately after intraluminal MCAO increased neurological deficits and brain infarct area (Wang et al., 2019). In Wistar rats, intraperitoneal LPS delivery (three injections of 50 μg/kg separated by 4 h) starting 24 h after intraluminal MCAO increased neurological deficits evaluated by Rotarod, grip strength, tape removal, and open-field tests; even though this study did not report a significant difference of infarct volume between groups (Yousuf et al., 2013; Atif et al., 2020). These results align with the results reported in humans that plasma endotoxin activity and stimulation of the pathogen-associated molecular pattern (PAMP) receptor TLR4 can contribute to unfavorable stroke outcomes *via* systemic inflammatory responses (Klimiec et al., 2016).

In the present study, intraperitoneal delivery of ketamine (10 mg/kg) reversed the detrimental effects of LPS on infarct volume and neurological deficits assessed by Clark score, cylinder, and open-field tests. In contrast, ketamine alone did not influence infarct volume or neurological deficits. Previously, low-dose ketamine (also 10 mg/kg) was shown to increase spontaneous locomotor activity, evaluated by the number of horizontal and vertical movements in open-field and forced swimming tests, of non-ischemic rats exposed to LPS (1 mg/kg) (Zhao J. et al., 2020). In ischemic C57BL/6 mice, repeated administration of ketamine (10 mg/kg) 30 min before MCAO and 24 h after MCAO reduced infarct volume and neurological deficits (Xiong et al., 2020). Neuroprotective effects of ketamine have also been reported in BALB/c mice following ketamine (25 mg/kg) administration 30 min post-MCAO (Shu et al., 2012). Differences in animal species and strains, injury models, drug doses, and delivery time points may explain diverging findings of these previous studies.

Mounting evidence gathered from literature reviews suggests a role of microglia and astrocytes as a source of pro-inflammatory and anti-inflammatory cytokines that worsen or improve neurological conditions (Lee et al., 2014; Lambertsen et al., 2019). In order to evaluate the underlying glial responses, we assessed the effects of LPS and ketamine by real-time qPCR, showing that LPS (4 mg/kg) significantly increased mRNA levels of the astrocytic marker *GFAP*, the pro-inflammatory microglia/macrophage marker *CD86*, and the anti-inflammatory microglia/macrophage marker *CD206* in ischemic brain tissue. Ketamine (10 mg/kg) did not alter *GFAP*, *CD86* or *CD206* mRNA levels. Post-ischemic LPS administration has previously been shown to increase astroglial GFAP immunoreactivity in mice exposed to MCAO (Sardari et al., 2020b) or in primary mouse astrocytes (Lin et al., 2016). LPS has recently been shown to increase CD86 expression in mice, as shown by positron emission tomography using the tracer abatacept (Taddio et al., 2021) and in microglia or macrophages *in vitro* (Ji et al., 2018; Liu et al., 2020). In CD57BL/6 mice exposed to MCAO, CD206^+^ microglia and macrophages gradually accumulate in ischemic brain tissue during the first-week post-stroke, subsequently replaced by CD86^+^ microglia and macrophages (Hu et al., 2012). *In vitro* studies show a time-dependent decrease in expression of M2 markers between 2 and 4 h after addition of LPS (10 ng/ml) to BV2 cell cultures (Wen et al., 2017). The same effect was also reported in other *in vitro* studies (Alves-Januzzi et al., 2017; Wu et al., 2019). Although we did not perform an in depth analysis of microglial markers and proinflammatory cytokines, we suggest that the increased CD206 expression *in vivo* in response to LPS exposure may result from the so-called compensatory anti-inflammatory response syndrome (CARS), aiming to overpower the LPS-associated acute inflammatory response (Maes and Carvalho, 2018).

Real-time qPCR showed that mRNA levels of the pro-inflammatory cytokine *IL-1*β and the anti-inflammatory cytokine *IL-10* were significantly increased by LPS (4 mg/kg). Ketamine (10 mg/kg) partly reversed the *IL-1*β and *IL-10* mRNA levels in LPS-treated mice, whereas ketamine alone did not affect them. Previous studies suggested that pro-inflammatory cytokines, such as IL-1β, mediate the behavioral effects of LPS. In male Sprague-Dawley rats, delayed LPS delivery (three times 50 μg/kg separated by 4 h) starting at 24 hpi significantly increased IL-1β levels after 48 and 72 h (Yousuf et al., 2013). In CB57BL/6 mice exposed to hypobaric hypoxia as a model of high altitude sickness, LPS (5 mg/kg) significantly increased brain IL-1β levels, which closely paralleled the development of blood-brain barrier breakdown, brain edema, and cognitive and motor deficits (Zhou et al., 2017). In non-ischemic Wistar rats, ketamine (10 mg/kg) has previously been shown to reduce elevated IL-1β levels induced by LPS (Zhao J. et al., 2020). LPS-induced IL-1β expression was significantly reduced by ketamine in astrocytic cultures (Zhang et al., 2012; Tanaka et al., 2013). In mononuclear cells, LPS was previously shown to increase IL-10 production (Takaono et al., 2002). Non-competitive NMDA antagonists, including the clinically administered memantine, modulate LPS-induced IL-10 expression (Simma et al., 2014). Ketamine (10 mg/kg) reduced LPS-induced (15 mg/kg) IL-10 expression in non-ischemic male Wistar rats (Gokcinar et al., 2013).

Taken together, our present study suggests a profound immunomodulatory effect of ketamine that mitigates the injury-exacerbating consequences of post-ischemic LPS-induced sepsis-like state. In view of the deleterious consequences of post-stroke infection, further studies on the underlying immunomodulatory actions of ketamine are warranted.

## Data Availability Statement

The original contributions presented in the study are included in the article/supplementary material, further inquiries can be directed to the corresponding author/s.

## Ethics Statement

The animal study was reviewed and approved by Research and Ethics Committee of the School of Biology, University of Tehran (IR.UT.SCIENCE.REC.1400.001).

## Author Contributions

MS and DH designed the study. GT performed animal experiments. MS performed molecular and Nissl staining. GT and MS analyzed data. MS, DH, GT, and HS drafted the manuscript. All authors listed have made a substantial, direct and intellectual contribution to the work, and approved it for publication.

## Conflict of Interest

The authors declare that the research was conducted in the absence of any commercial or financial relationships that could be construed as a potential conflict of interest.

## Publisher’s Note

All claims expressed in this article are solely those of the authors and do not necessarily represent those of their affiliated organizations, or those of the publisher, the editors and the reviewers. Any product that may be evaluated in this article, or claim that may be made by its manufacturer, is not guaranteed or endorsed by the publisher.

## Footnotes

1
https://blast.ncbi.nlm.nih.gov
